# Complete mitochondrial genomes of five subspecies of the Eurasian magpie *Pica pica*, obtained with Oxford Nanopore MinION, and their interpretation regarding intraspecific taxonomy

**DOI:** 10.1080/23802359.2020.1838354

**Published:** 2020-11-20

**Authors:** Alexey P. Kryukov, Liudmila N. Spiridonova, Alexey P. Tyunin, Kirill A. Kryukov, Beatriz A. Dorda

**Affiliations:** a Federal Scientific Center of the East Asia Terrestrial Biodiversity (FSCEATB), Far Eastern Branch of the Russian Academy of Sciences, Vladivostok, Russia; b National Institute of Genetics, Mishima, Japan; c National Museum of Natural Science (MNCN-CSIC), Madrid, Spain

**Keywords:** Mitochondrial genome, Eurasian magpie, *Pica pica*, *Pica serica*, Corvidae

## Abstract

The complete mitochondrial (mt) genomes of five subspecies of the Eurasian (Common) magpie *Pica pica* were determined for the first time. Lengths of the circular genomes comprise 13 protein-coding genes, two rRNA genes (for 12S rRNA and 16S rRNA), 22 tRNA genes, and the non-coding control region (CR). Gene content and lengths of the genomes (16,936–16,945 bp) are similar to typical vertebrate mt genomes. The subspecies studied differs by several single substitutions and indels, especially in the CR. The phylogenetic tree based on complete mt genomes shows a deep divergence of the two groups of subspecies which supports the proposed division into two distinct species: *P. pica* and *P. serica*.

Complete mitochondrial (mt) genomes are still rarely explored in intraspecies systematics. Taxonomy, species borders, and intraspecies structure of the Eurasian (Common) magpie *Pica pica* L. (Passeriformes, Corvidae) are a matter of debate. It is widespread in the Holarctic. For the Palearctic taxa, taxonomy varies from accepting a single species with 12 subspecies (Madge and Burn 1999; Kryukov et al. 2017) to division into four species (Song et al. 2018). Only one complete mt genome of *P. pica* has been reported previously (GenBank HQ915867). Here, we describe the complete mt genomes of five subspecies (sensu Kryukov et al. 2017) of magpie and use them for assessing intraspecies relationships.

Tissue (blood or feather) samples were collected: *P. p. camtschatica* in Kamchatka Peninsula, Russia (54.30N, 156.00E); *P. p. jankowskii* in Primorsky Krai, Russia (43.84N, 131.86E); *P. p. fennorum* in Moscow region, Russia (55.75N, 38.01E); *P. p. leucoptera* in Mongolia (48.45N, 115.35E); *P. p. melanotos* in Buitrago del Lozoya, Spain (40.99N, 3.64W). The samples are stored in the tissue and DNA collections of FSCEATB, Russia (#2320 for *camtschatica*, #2205 for *jankowskii*, #1215 for *fennorum*, and #2385 for *leucoptera*), and MNCN, Spain (#2804 for *melanotos*). DNA was extracted with DNeasy Blood and Tissue kit (Qiagen, Hilden, Germany). Two primer pairs were designed for the amplification of two overlapping fragments: mtPica-66F (GACAAAAGACTTAGTCCTAACCTTACTGTT) and mtPica-7010R (GTGGTTTATGCGGTTGGCTTGAA) for a ∼7-kb fragment; mtPica-5359F (CCTCTGTAAAAAGGACTACAGCCTA) and mtPica-68R (AGTAAGGTTAGGACTAAGTCTTTTG) for a ∼11-kb fragment. The amplicons obtained were individually tagged with barcode sequences and sequenced using the Oxford Nanopore MinION with Flow Cell R10 and the protocol ‘1d-native-barcoding-genomic-dna-NBE_9065_v109_revI_23May2018.’ The reads were aligned one by one to the reference genome HQ915867 with AliView v. 1.17.1 (Larsson 2014) and consensus sequences were completed using Consensus Tool (http://kirr.dyndns.org/bio/consensus/). Annotation was performed with MITOS (Bernt et al. 2013).

The assembled genomes are 16,936–16,945 bp in length and each contains 37 genes comprising 13 protein-coding genes, two rRNA genes (for 12S rRNA and 16S rRNA), and 22 tRNA genes. The control region (CR) is located between tRNA-Glu and tRNA-Phe genes and has a length of 1351–1362 bp. The H- and L-strands encode 28 and nine genes, respectively. The gene arrangement is the same in all genomes studied. GC content of the new genomes is 43.3–43.5%. Proportions of A, T, C, and G in subspecies have the following ranges (in %): 31–31.1, 25.4–25.6, 29.2–29.5, and 14.0–14.1, respectively.

For illustrating phylogenetic relations of the complete mt genomes obtained, we constructed a maximum-likelihood (ML) tree (Figure 1) using MEGA X (Kumar et al. 2018).

**Figure 1.**
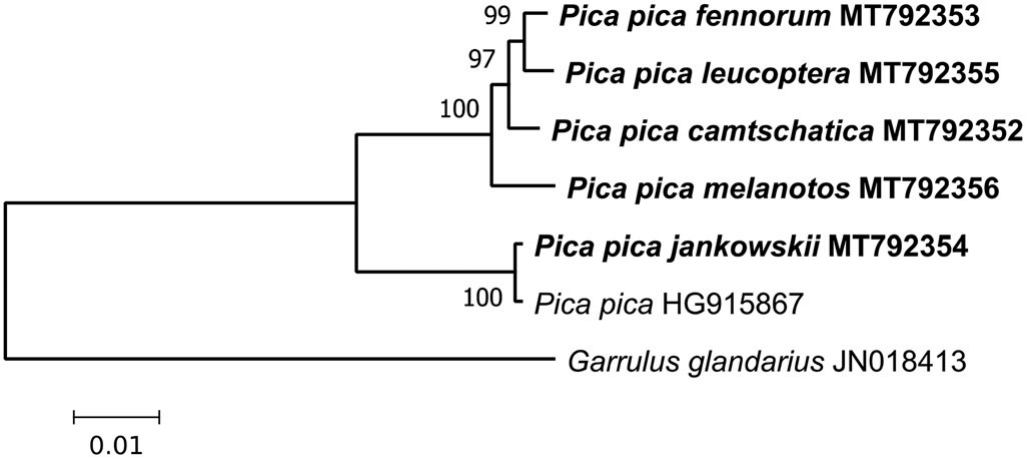
Maximum-likelihood phylogenetic tree for the five newly obtained complete mitochondrial genomes of *Pica pica* s.l., and the previously determined sequence of *Pica pica* (GenBank HQ915867)*. Garrulus glandarius* (JN018413) is used as outgroup. Numbers at nodes are bootstrap support values in %.

The western group of subspecies includes *fennorum*, *melanotos*, *leucoptera*, as well as geographically distant *camtschatica*. *Jankowskii* is nearly identical to HQ915867. The two clades are well differentiated by *p*-distance of .039 on average. In general, this is consistent with the previous results obtained using individual genes: *cytB* and CR (Kryukov et al. 2004, 2017; Haring et al. 2007); *cytB*, *ND2*, and two nuclear introns (Song et al. 2018). This degree of divergence supports classifying the two clades as separate species.

The subspecies name of HQ915867 is not mentioned in its GenBank entry. Its near identity to *jankowskii* suggests that it could belong to *jankowskii* or a closely related subspecies, such as *serica* or *anderssoni*. Kryukov et al. (2017) showed close affinity of *serica* and *jankowskii*, while Song et al. (2018) showed near identity of *serica* and *anderssoni*, and suggested grouping them under *Pica serica* species. Based on our complete mt genome data, we support classifying *Pica serica* as a species, distinct from *P. pica* (the western clade). *Pica serica* species may include *jankowskii*, *serica*, and *anderssoni* subspecies.

Continuation of genomic studies is necessary for clarifying taxonomy of the remaining subspecies.

## Data Availability

The data that support the findings of this study are openly available in the National Center for Biotechnology Information database (NCBI/GenBank) at https://www.ncbi.nlm.nih.gov/, reference numbers MT792352, MT792353, MT792354, MT792355, and MT792356.
